# The battle for iron in enteric infections

**DOI:** 10.1111/imm.13236

**Published:** 2020-08-11

**Authors:** Ana Sousa Gerós, Alison Simmons, Hal Drakesmith, Anna Aulicino, Joe N. Frost

**Affiliations:** ^1^ MRC Human Immunology Unit Weatherall Institute of Molecular Medicine University of Oxford Oxford UK; ^2^ Translational Gastroenterology Unit John Radcliffe Hospital Oxford UK

**Keywords:** *Clostridia*, gut microbiota, iron, nutritional immunity, *Salmonella*

## Abstract

Iron is an essential element for almost all living organisms, but can be extremely toxic in high concentrations. All organisms must therefore employ homeostatic mechanisms to finely regulate iron uptake, usage and storage in the face of dynamic environmental conditions. The critical step in mammalian systemic iron homeostasis is the fine regulation of dietary iron absorption. However, as the gastrointestinal system is also home to >10^14^ bacteria, all of which engage in their own programmes of iron homeostasis, the gut represents an anatomical location where the inter‐kingdom fight for iron is never‐ending. Here, we explore the molecular mechanisms of, and interactions between, host and bacterial iron homeostasis in the gastrointestinal tract. We first detail how mammalian systemic and cellular iron homeostasis influences gastrointestinal iron availability. We then focus on two important human pathogens, *Salmonella* and *Clostridia*; despite their differences, they exemplify how a bacterial pathogen must navigate and exploit this web of iron homeostasis interactions to avoid host nutritional immunity and replicate successfully. We then reciprocally explore how iron availability interacts with the gastrointestinal microbiota, and the consequences of this on mammalian physiology and pathogen iron acquisition. Finally, we address how understanding the battle for iron in the gastrointestinal tract might inform clinical practice and inspire new treatments for important diseases.

## Graphical abstract

Intestinal absorption of dietary iron is the key step regulating systemic iron homeostasis, but the human gut is also home to 100 trillion bacteria, which have their own, highly variable, iron requirements. Here, we explore molecular mechanisms underlying competition for this essential nutrient, focussing on salmonella, clostridia, and effects of iron acquisition on physiology during enteric infections. We discuss how understanding the battle for iron in the gut impacts on treatments for common intestinal inflammatory disorders.

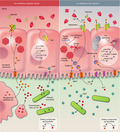

**Abbreviations**
aTf: apo‐transferrin
DCYTB: duodenal cytochrome *b*
DMT1 (or NRAMP2): divalent metal transporter protein 1
DtxR: diphtheria toxin repressor
Fe‐S: iron‐sulphur
FPN (or SLC40A1): ferroportin
Ft: ferritin
FtH: ferritin heavy subunits
FtL: ferritin light subunits
Fur: ferric uptake regulator
HAMP: hepcidin
HIF2*α*: hypoxia‐inducible factor 2; *α*
hTf: holo‐transferrin
IRP: iron regulatory proteins
LCN2: lipocalin 2
Lf: lactoferrin
NRAMP1 (or SLC11A1): natural resistance‐associated macrophage protein 1
ROS: reactive oxygen species
Tf: transferrin
TfR1 (or CD71): transferrin receptor 1

## Iron in biological systems

Iron plays a central role in the biochemistry of nearly all life forms, where it is found within haem and inorganic compounds.^1^, ^2^ Inorganic iron may occur as ferrous (Fe^2+^) and ferric (Fe^3+^) iron, the reduced and oxidized states of the element, respectively.^3^ The ubiquity of iron as a cofactor in biomolecules is in part due to its versatile chemistry. Iron has a large number of accessible oxidation states, allowing it to shuttle electrons, catalyse reactions and form a wide range of co‐ordination complexes. Furthermore, iron was highly bioavailable during the early stages of the evolution of life, potentially rendering biochemistry irreversibly ‘addicted’ to iron as a metallic cofactor.^4^ In the human body, iron occurs in inorganic compounds (e.g. iron oxalate, iron citrate, iron phytate, iron sulphate) and in iron‐containing proteins. These can be categorized as (i) haemoproteins, (ii) iron–sulphur (Fe–S) cluster proteins, and (iii) non‐haem, non‐Fe–S, iron‐containing proteins. A multitude of crucial biological processes in eukaryotes and prokaryotes depend on iron‐containing proteins: DNA synthesis; metabolic energy production; biosynthesis of organic compounds, such as hormones; regulation of gene expression by histone and DNA demethylases; oxygen transport; and production of and defence against free radicals.^2^, ^5^, ^6^, ^7^, ^8^

### Ingestion, absorption and intracellular handling of iron in humans

In mammals, excretion of iron is not extensively regulated; instead, iron homeostasis is achieved mainly by regulating intestinal absorption in the duodenum,^9^ and possibly in the colon to some extent.^10^ On average, humans ingest 20–25 mg of iron/day, the majority of which is inorganic iron (85%–90%), followed by haem‐bound iron (10%–15%). However, only ~ 1 mg iron is absorbed per day, because of both poor iron bioavailability and tightly regulated iron homeostasis.^9^, ^11^

Of the two oxidation states that inorganic iron may take, Fe^3+^ is the most stable in physiological pH and oxygen conditions, and also the most commonly found in dietary products.^3^ However, inorganic iron uptake from the gastrointestinal lumen by enterocytes depends upon the divalent metal transporter protein 1 (DMT1 or NRAMP2), which is not able to import Fe^3+^.^12^, ^13^, ^14^ The low pH of the stomach lumen reduces a significant fraction of Fe^3+^ to Fe^2+^, thereby facilitating its solubility and absorption. However, increased pH in the small intestine results in the oxidation and precipitation of a large fraction of dietary iron, rendering it inaccessible. To overcome the low availability of Fe^2+^, duodenal cytochrome *b* (DCYTB) enzymes that are present within the apical membrane of enterocytes are able to reduce any soluble Fe^3+^ to Fe^2+^ before DMT1‐mediated Fe^2+^ import.^14^, ^15^ Organic molecules such as dietary vitamin C and microbe‐produced short‐chain fatty acids can also reduce iron and increase its bioavailability.^11^, ^16^, ^17^ Finally, the activity of the Na^+^/H^+^ exchanger NHE3, which exports H^+^ from the enterocyte and generates an electrochemical gradient of H^+^ into the cell, facilitates the symport of Fe^2+^ and H^+^ by DMT1.^18^

Despite haem’s small contribution to the total amount of iron ingested, it still accounts for over 40% of the daily absorbed iron, mainly as a result of its significantly higher bioavailability when compared with inorganic iron.^11^ The mechanism of haem uptake into enterocytes is unclear, but may involve the haem transporter HRG1, followed by intracellular degradation by the haem oxygenase HMOX1 to finally release Fe^2+^.^19^, ^20^

Enterocytes have a relatively short lifespan (~ 4 days in humans), and, once sloughed off, all of their intracellular content is lost into the gut lumen. Therefore, in order to be absorbed, iron must promptly cross the basolateral membrane of enterocytes to reach the circulation. Cellular iron export – and, therefore, gastrointestinal absorption of iron – is uniquely mediated by the iron transporter ferroportin (FPN or SLC40A1).^21^, ^22^, ^23^, ^24^ As discussed in the next section, regulation of ferroportin‐mediated iron export is central to systemic iron homeostasis. Immediately after crossing the basolateral membrane, Fe^2+^ is oxidized to Fe^3+^ by hephaestin, a multicopper oxidase co‐localized with FPN.^25^, ^26^ FPN is also found within the membranes of intracellular vesicles – including pathogen‐containing phagosomes,^27^ particularly in macrophages.

Upon entering the circulation, most Fe^3+^ is rapidly bound to transferrin (Tf), the main form of circulating iron. Uptake of iron‐bound Tf – holo‐transferrin (hTf) – complexes by transferrin receptor 1 (TfR1 or CD71) is also the dominant mechanism of cellular iron acquisition by many cells and tissues.^28^ When bound together, TfR1–hTf complexes are endocytosed, releasing Fe^3+^ in weakly acidic endosomes. Within these vesicles, ferrireductases, such as six‐transmembrane epithelial of the prostate 3, convert Fe^3+^ into Fe^2+^ once again.^29^ TfR1 remains bound to the iron‐free Tf – apo‐transferrin (aTf) – and the complex returns to the cell surface. There, aTf is released back into the circulation, where it may capture iron again.^30^

In endosomes, Fe^2+^ enters the cytosol via DMT1. Free iron in the cytosol might partake in dangerous biochemical reactions; however, the factors that control trafficking of the cellular labile iron pool are only beginning to be understood. The moonlighting RNA‐binding proteins PCBP1 and PCBP2 have been proposed to play a role in chaperoning cytoplasmic iron.^31^, ^32^ Labile iron can then be: (i) placed into cytosolic iron‐containing proteins; (ii) transported to the mitochondria and incorporated into haem and Fe–S clusters;^2^, ^33^ (iii) sequestered inside ferritin (Ft); or (iv) exported to the extracellular space by FPN.^30^ Nanocages of Ft, formed from 24 light (FtL) and heavy (FtH) subunits, provide a long‐term storage site for iron, shielding the cell from potential redox activity of free iron.^34^ During iron deficiency, iron can be liberated from the Ft cage by a specific form of autophagy – ferritinophagy – mediated by the adaptor protein NCOA4.^35^, ^36^

### Host systemic iron metabolism and regulation of iron absorption

Every day 200 billion erythrocytes are degraded by reticuloendothelial macrophages in the liver and spleen; replacing these red blood cells has been estimated to require 2 × 10^15^ iron atoms or 20 mg of iron.^30^ Hence, recycling of erythrocyte‐derived iron via reticuloendothelial macrophages is essential for organism survival.^30^ However, every day around 1 mg of iron is lost from the body in an unregulated way – predominantly via the sloughing off of epithelial cells – and this must be compensated by finely regulated intestinal iron absorption to keep total body iron content in balance.

The key to systemic iron homeostasis lies with hepcidin (HAMP) regulation.^37^ HAMP is a 25‐amino‐acid hormone protein mainly produced by liver hepatocytes, and its production is induced by iron loading.^38^ HAMP‐deficient mice and humans present with a severe iron loading phenotype, and injection of synthetic HAMP decreases serum iron concentrations.^39^, ^40^, ^41^ Similarly, haemochromatosis is a genetic disease characterized by mutations in genes involved in iron homeostasis (*HFE*,* HJV* and* TFR2)*. The proteins encoded by these genes play essential roles in controlling HAMP synthesis induced by intracellular and circulating iron stores, via the bone morphogenetic protein/SMAD signalling pathway.^30^ In haemochromatosis, intestinal iron absorption is disconnected from systemic iron availability, resulting in potentially lethal iron loading and susceptibility to fatal siderophilic bacterial infections, which exemplify the importance of regulating intestinal iron absorption.^42^, ^43^

**Figure 1:**
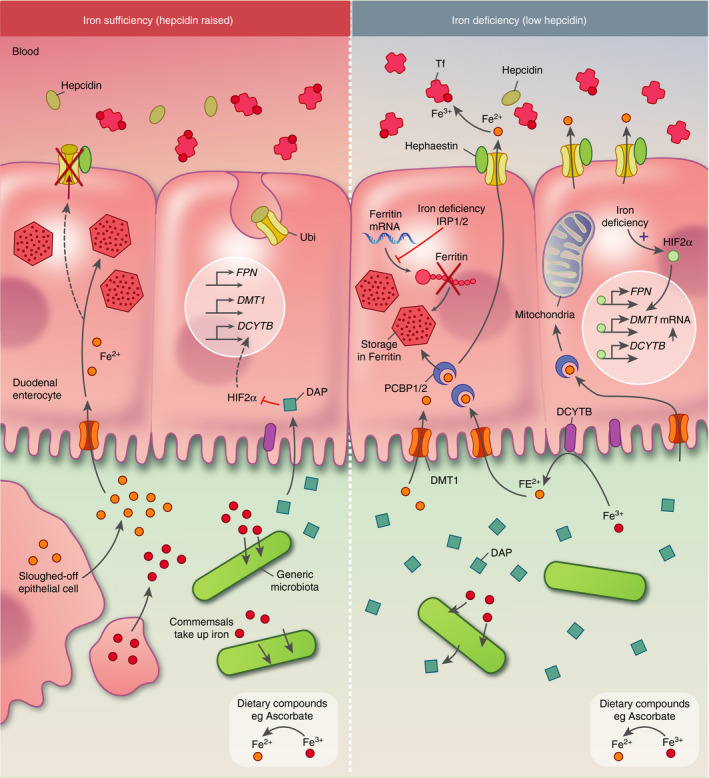
Intestinal iron homeostasis. Fe^3+^ is reduced to Fe^2+^ by duodenal cytochrome *b* (DCYTB) on the apical membrane of duodenal enterocytes and dietary compounds. Fe^2+^ is taken up by enterocytes through divalent metal transporter protein 1 (DMT1). Inside the cell, iron is partitioned into various compartments, in part through chaperoning by proteins such as PCBP1/2. Iron may be used for biosynthesis of Fe‐S clusters or haem in the mitochondria, or stored as ferritin in the mitochondria and cytosol. Cytoplasmic Fe^2+^ is exported out of the enterocyte into the blood through ferroportin (FPN), where it is oxidized to Fe^3+^ by hephaestin and bound by transferrin. In conditions of iron deficiency, hypoxia‐inducible factor 2*α* (HIF2*α*) is stabilized, promoting the transcription of *FPN*, *DMT1* and *DCYTB*, facilitating further iron uptake. Enterocyte iron deficiency also results in increased RNA‐binding activity of iron regulatory proteins 1 and 2 (IRP1/2), which represses the translation of ferritin, reducing iron sequestration inside the enterocyte. Local iron homeostasis is further modulated by the microbiota, in conditions of iron deficiency microbial species such as *Bifidobacterium* generate small organic molecules such as 1,3‐diaminopropane (DAP) which repress HIF2*α* and reduce transcription of the iron transporters. The production of HIF2*α* inhibitors by the gut microbiota may be a mechanism by which the microbiota competes with the host for luminal iron. Iron homeostasis is systemically regulated by hepcidin (HAMP) production in the liver; when iron levels are systemically raised, HAMP is produced. HAMP binds to, occludes and stimulates the degradation of FPN preventing iron export from the enterocyte and therefore limiting further iron uptake from the diet. In the absence of iron export, enterocyte iron will be stored in ferritin. The iron concentration in the intestinal lumen is set by the iron requirements of the gastrointestinal microbiota, iron uptake into enterocytes via DMT1 and release of enterocyte iron stores back into the lumen by epithelial sloughing.

HAMP regulates systemic iron metabolism through its capacity to bind, occlude and induce the degradation of FPN on the surface of reticuloendothelial macrophages and duodenal enterocytes.^44^, ^45^ In conditions of iron accumulation, the production of HAMP is stimulated, and, as a result, iron is subsequently trapped within enterocytes and macrophages, respectively inhibiting iron export to circulation or preventing recycling of iron derived from red blood cells.^9^ This acutely decreases serum iron and reduces dietary absorption to prevent further iron loading. Conversely, in conditions of iron deficiency, HAMP expression is down‐regulated and surface expression of FPN is maintained, allowing iron export into the circulation and thereby replenishing overall iron levels (Fig. 1).

In addition to systemic regulation of iron flux out of the duodenal epithelia by HAMP, the organismal response to iron deficiency is also critically dependent on local signal integration. Iron deficiency results in stabilization of duodenal Hypoxia‐Inducible Factor 2*α* (HIF2*α*), which in turn transcriptionally up‐regulates expression of *DMT1*, *DCYTB* and *FPN* in enterocytes.^46^, ^47^, ^48^ Mice deficient in enterocyte HIF2*α* tolerate iron deficiency poorly, rapidly becoming anaemic as they cannot maximize iron flux through the duodenal enterocyte. Control of FPN‐mediated iron efflux by HAMP has been proposed to modulate enterocyte iron content and therefore HIF2*α*‐regulated iron uptake, indicating how both systemic and local iron homeostasis are coupled^49^ (Fig. 1).

Intracellular iron homeostasis in mammals is also controlled by two iron regulatory proteins (IRP1 and IRP2), which sense iron and post‐transcriptionally regulate the expression of a number of iron homeostatic genes.^50^ As mentioned, iron can be stored in Ft, instead of exported out of the cell via FPN. IRPs negatively regulate Ft translation to prevent inappropriate iron sequestration in enterocytes.^51^, ^52^ Hence, multiple regulatory mechanisms exist within and acting upon duodenal enterocytes to regulate flux into, through and out of the enterocyte into the circulation. It is important to note that any mechanisms that reduce intestinal iron uptake will in turn relatively increase availability of iron in the gastrointestinal tract.

### Bacteria and iron: sensing and homeostasis, not just thievery

Iron is an essential element for the growth of most bacteria,^53^, ^54^ which rely almost exclusively on Fe^2+^ uptake mechanisms. However, due to the low solubility of Fe^2+^ over the soluble Fe^3+^, iron is frequently a limiting nutrient. The capacity to acquire sufficient iron to support proliferation, in the presence of iron‐limiting host defence mechanisms, is an important determinant of pathogenicity. Iron deprivation acts as a sensory cue in bacterial pathogens, triggering the coordinated regulation of iron acquisition and virulence genes. Bacterial responses to iron depletion are largely controlled by two families of highly conserved iron‐responsive regulators: the ferric uptake regulator (Fur) superfamily^55^ – mostly expressed in Gram‐negative bacteria – and the diphtheria toxin repressor family (DtxR) – occurring in many species of Gram‐positive bacteria.^56^ Despite their dissimilarities, Fur and DtxR‐like proteins have equivalent domain architectures.^56^ Moreover, both metalloproteins function similarly, allowing/repressing transcription of target genes and modulating the expression of virulence factors such as toxin secretion, production of adhesins, formation of biofilms and regulation of quorum sensing.^57^, ^58^

Fur and DtxR both bear a DNA‐binding domain as well as a metal co‐repressor binding site.^59^, ^60^ In iron‐rich conditions, these metalloproteins bind Fe^2+^, undergoing a conformational change that favours their binding to specific motifs within the promoter region of target genes.^60^ Binding of Fur–Fe^2+^ and DtxR–Fe^2+^ upstream of iron uptake and storage operons/genes inhibits their transcription under iron‐replete conditions.^56^, ^61^ Conversely, in iron deficiency, Fe^2+^ dissociates from Fur/DtxR, which unbind from DNA, allowing the transcription of essential iron harvesting genes.^62^ Indeed, most iron acquisition strategies employed by bacterial pathogens are regulated by members of the Fur or DtxR superfamilies.

In this review, we particularly focus on two instructive gastrointestinal pathogens in the context of host–pathogen iron interactions. *Salmonella enterica* serovar Typhimurium (*S*. Typhimurium) is a Gram‐negative facultative intracellular pathogen, which commonly causes localized gastrointestinal disease. In turn, despite a complex and multifaceted host immune response,^63^ the typhoidal strains *Salmonella* Typhi (*S*. Typhi) and Paratyphi – as well as other specific non‐typhoidal serovars – have the potential to breach the mucosa and cause severe systemic infection, through initial intracellular infection of macrophages.^64^, ^65^, ^66^ *Clostridium difficile* is a Gram‐positive extracellular pathogen, which remains localized to the gastrointestinal tract but can cause severe toxin‐mediated gastrointestinal pathology, with a mortality rate of up to 6%.^67^, ^68^

### Iron homeostasis in *Salmonella*

Approximately 7% of the *S*. Typhimurium genome is directly or indirectly regulated by iron,^69^ which highlights the importance of iron to *Salmonella*. Central to iron homeostasis in *Salmonella* is Fur, which controls essential mechanisms of iron uptake and storage.^61^, ^62^ Furthermore, indirect regulation by the Fur‐inhibited repressor RyhB sRNA suppresses expression of non‐essential iron‐requiring proteins during iron depletion.^62^, ^70^, ^71^, ^72^ Fur also attenuates iron overload by controlling the production of bacterial Ft, which stores iron and protects the cell against iron‐catalysed reactive oxygen species (ROS).^73^, ^74^ Fur‐independent iron‐driven changes in gene expression have also been observed;^69^ for example, extracellular iron concentration is one of the stimuli of the two‐component sensing pathway PhoQ‐PhoP.^75^

Besides controlling the expression of genes directly involved in iron homeostasis, Fur regulation extends to other fundamental pathways, such as nitrate/nitrite respiration,^76^ acid tolerance response,^77^ and virulence – including the Type 3 secretion system encoded by *Salmonella* pathogenicity island‐1.^78^, ^79^, ^80^ Fur‐mediated regulation of virulence genes probably underpins the observed enhancement of *Salmonella* virulence in iron‐supplemented conditions.^81^ Fur can also itself be regulated by the peroxide sensor OxyR, with ROS‐mediated induction of Fur probably preventing oxidation of biomolecules by reducing intracellular iron concentration and subsequent formation of hydroxyl radicals.^82^

Overall, iron sensing via Fur and RyhB sRNA is essential for *S*. Typhi^83^ and *S*. Typhimurium^84^ infection. This finding underpins the multifaceted role that Fur plays in the survival of *Salmonella* in the host, by integrating iron handling, ROS, virulence and other fundamental signalling pathways. Therefore, it may be inferred that *Salmonella* harnesses iron availability to extrapolate information on its wider external environment and guide its behaviour.

In addition to Fur‐mediated iron sensing, haem biosynthesis is also exquisitely regulated, with haem negatively regulating the activity of the rate‐limiting biosynthetic enzyme HemA.^85^, ^86^ Haem compounds are crucial cofactors for many bacterial cytochromes and catalases. In *S*. Typhimurium, the haem biosynthetic pathway is essential both for oxidative respiration and for protection against toxic oxygen intermediates (such as hydrogen peroxide).^87^, ^88^ It also branches out into two distinct pathways related to the synthesis of sirohaem (a substrate in cysteine biosynthesis) and of cobalamin (or vitamin B12, a cofactor to many different enzymes).^87^ It was previously shown that *S*. Typhimurium *hemA* mutants were avirulent in mice,^89^ and their exposure to hydrogen peroxide resulted in extensive iron‐mediated DNA damage and cell death.^87^

### Response to iron deficiency in *C. difficile*

Similarly to *Salmonella*, exposure to low iron evokes a stress response in *C. difficile,* with many of the differentially expressed genes exhibiting binding sites for Fur.^90^ This results in altered expression of iron transporters – thus increasing iron uptake – and a metabolic switch, by suppressing the activity of metabolic pathways requiring iron‐containing proteins and favouring alternative mechanisms. Specifically, glucose metabolism by pyruvate formate‐lyase, formate dehydrogenase and [FeFe]‐hydrogenase, ferredoxin‐dependent amino acid fermentation, and cell motility are all suppressed by iron deficiency. *Clostridium difficile* also significantly changes the composition of its cell wall in response to iron deficiency, presumably to protect itself from other microorganisms, antibiotics or host immune responses.^91^ Moreover, iron depletion revealed significant up‐regulation of *C. difficile* genes associated with virulence, including polyamine and histidine biosynthesis and uptake, as well as several flagella‐associated genes, which represent well‐known factors involved in adherence.^92^ The diverse changes in gene expression mediated by Fur underpin how sensing and responding to iron availability is synonymous with sensing the diverse environments to which a pathogen is exposed as it moves through the host.

### The host modulates systemic iron metabolism to limit extracellular iron availability

Withholding of nutrients, particularly iron, from invading microorganisms has long been understood to play a key role in innate immune responses to infection.^93^ IRP, Ft and the siderophore‐binding protein lipocalin 2 (LCN2) play pivotal roles in preventing and regulating intracellular infection, underscoring iron’s key role in determining the outcome of pathogen invasion.^94^, ^95^

**Figure 2:**
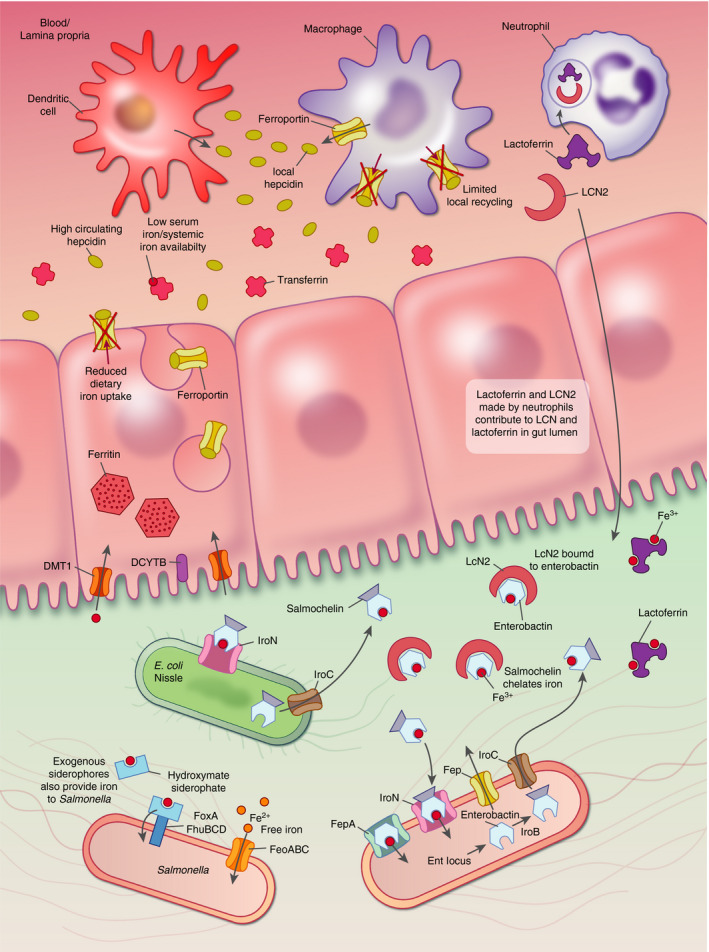
Intestinal iron homeostasis during inflammation. Systemic inflammation increases hepcidin (HAMP) production by the liver, reducing iron absorption by the enterocyte and preventing iron recycling out of reticuloendothelial macrophages. Serum iron levels drop, and iron is sequestered within enterocytes. Furthermore, locally within the lamina propria, HAMP produced by dendritic cells has been shown to suppress ferroportin (FPN) on gut macrophages and regulate local iron availability during inflammation. In the gut lumen, pathogenic bacteria such as *Salmonella* can take up Fe^2+^ directly (via systems such as FeoABC) or produce siderophores (such as enterobactins and salmochelins) to facilitate uptake of Fe^3+^. To sequester iron in the gut lumen, enterocytes and immune cells, particularly neutrophils, produce iron‐sequestering proteins such as Fe^3+^‐binding lactoferrin (Lf) and enterobactin‐targeting lipocalin 2 (LCN2). Salmochelin is not bound by LCN2 and allows *Salmonella* to acquire iron even in inflammatory situations. Some commensals, such as *Escherichia coli* strain Nissle, disrupt iron acquisition by *Salmonella* by competing for iron through the production of salmochelin and expression IroN, its uptake receptor.

Additionally, and besides controlling iron homeostasis, the iron regulatory hormone HAMP can also reprogramme systemic iron distribution to protect against infection.^96^ HAMP was first identified as a liver‐produced relative of the defensin antimicrobial peptide family.^97^ During inflammation, HAMP is induced in hepatocytes alongside a battery of other acute‐phase proteins, downstream of interleukin‐6 and toll‐like receptor signalling‐associated pathways.^98^ This increased production of HAMP during inflammation, and the subsequent block on iron export into the serum, drives the commonly observed hypoferraemia of inflammation (Fig. 2). Serum iron deficiency in humans is protective against the growth of some bacteria,^99^ and HAMP plays a role in preventing uncontrolled systemic infection with extracellular bacteria – including *Vibrio vulnificus*, *Yersinia enterocolitica* and some pathogenic *Escherichia coli* variants.^100^, ^101^, ^102^, ^103^ However, many bacteria are unaffected by host responses elicited by endogenous (or therapeutic) HAMP, which may be partly explained by their capacity to obtain iron from diverse sources *in vivo*, such as haem.^100^

In contrast to its clear inhibitory activity against some extracellular pathogens, HAMP‐mediated hypoferraemia may promote intracellular bacterial growth in macrophages.^104^, ^105^ HAMP‐mediated degradation of FPN in infected macrophages greatly reduces iron export from the cytosol. Recent work suggests that HAMP can induce the degradation of phagosomal membrane FPN as well as FPN in the plasma membrane.^27^ Reduced inflow of cytosolic iron decreases intra‐vacuolar iron, so reducing iron‐catalysed ROS production and originating a more amenable intra‐phagosomal environment, which facilitates *Salmonella* survival and replication.^27^

Although hepatic HAMP is necessary to control systemic iron homeostasis,^106^ transcription of *HAMP* has been observed in a range of other cell types. Recently, in an experimental murine dextran sodium sulphate‐driven colitis, HAMP production by dendritic cells was proposed to drive local iron sequestration in colonic myeloid cells, limiting increases in free iron driven by loss of barrier integrity and tissue damage. Failure to sequester iron locally resulted in dysbiosis, increased microbial translocation across the mucosa, persistent gut inflammation and failure to recover weight^107^ (Fig. 2). Independent work proposed a role for the Spi‐C transcription factor and FPN‐expressing macrophages in protection against dextran sodium sulphate‐driven colitis.^108^ HAMP produced locally by keratinocytes may play a role in skin infection, in part by modulating the local innate immune response and neutrophil recruitment.^109^ These results pave the way for future work exploring the role of HAMP as a local regulator of iron–immune cell–microorganism interactions.

Another crucial host defence strategy lies on the expression of the natural resistance‐associated macrophage protein 1 (NRAMP1, or SLC11A1), a divalent metal ion transporter expressed on the membranes of phagolysosomes of phagocytic cells.^110^ NRAMP1 polymorphisms can modulate the sensitivity of humans and mice to intracellular bacterial infections.^111^ NRAMP1 exerts its antimicrobial effects by two distinct mechanisms. First, it can modulate the phagolysosomal metal composition and the access of pathogens to important micronutrients, such as Fe^2+^ and Mn^2+^.^112^, ^113^ Iron depletion inhibits bacterial growth and pathogenicity, and low manganese levels decrease the resistance of microbes to oxidative stress.^114^ Recent results suggest manganese deprivation is the major NRAMP1‐mediated *Salmonella* resistance mechanism. Second, NRAMP1 activates pro‐inflammatory immune pathways and antimicrobial effector mechanisms, resulting in increased production of reactive oxygen intermediates and reactive nitrogen intermediates.^115^

## Host iron‐binding proteins and mechanisms of bacterial iron uptake

It is instructive to view bacterial iron acquisition mechanisms through the lens of counteracting host nutritional immune defences. Broadly, the mechanisms for iron uptake adopted by pathogenic bacteria include: (i) uptake of free inorganic iron, facilitated by reductases and associated Fe^2+^ permeases; (ii) acquisition of Tf‐ and lactoferrin (Lf) ‐bound Fe^3+^, through direct or siderophore‐mediated uptake, and (iii) extraction and capture of haem‐iron from host haemoproteins, through haemolysin and/or haemophore secretion and specific cell surface‐associated receptors.^116^

### Uptake of free inorganic iron

*Salmonella* captures free Fe^2+^ by a number of transporters, primarily the FeoABC system.^117^ An *S*. Typhimurium mutant for FeoB was shown to be outcompeted by the wild‐type during mixed colonization of the mouse intestine.^118^, ^119^ Availability of Fe^2+^ *in vivo* might be increased by host‐ and bacteria‐encoded ferric reductases.^120^ However, recently, the host Mn^2+^/Zn^2+^‐chelating protein calprotectin was proposed to limit bacterial iron acquisition through its capacity to bind Fe^2+^.^121^ *Clostridium difficile* expresses many genes – most of which are Fur‐regulated – which encode paralogous Feo transport systems. From these, the operon *feo1* was consistently shown to be highly induced under iron‐depleted conditions.^90^, ^91^, ^92^ In addition to Feo systems, other Fur‐regulated putative iron transporters might also import free Fe^2+^ from the external milieu, such as proteins similar to P‐type cation transporters, and the low‐affinity zinc transporter ZupT.^90^, ^91^

### Direct and siderophore mediated uptake of Tf‐ and Lf‐bound Fe^3+^

Tf and Lf are the dominant host iron‐binding proteins in blood/tissue fluid and exocrine secretions, respectively. Both target free Fe^3+^,^122^ and can therefore reduce its availability. Unsurprisingly, many pathogens are capable of circumventing iron withholding by Tf and Lf by directly taking up and degrading these glycoproteins. Primate Tf shows evidence of positive evolutionary selection at amino acid residues bound by bacterial Tf receptors, indicating the evolutionary pressure on the host to deprive pathogens of iron and the importance of Tf for host defence.^123^

As an alternative to directly taking up Tf or Lf, some bacteria produce siderophores; these are small molecules with extremely high affinity for Fe^3+^, frequently employed as a tool to hijack iron from host glycoproteins (such as Lf, Tf and Ft), and to scavenge residual free iron from the external milieu. Siderophores are categorized based on the ferric‐binding moiety, and include carboxylates, catecholates, hydroxamates and phenolates.^124^ Siderophores are particularly important for the pathogenesis of *Salmonella*, which – like many *Enterobacteriaceae* – synthesizes and utilizes the siderophore enterobactin through the Fep operon. In addition to supplying iron for growth, enterobactin has been proposed to inhibit iron‐dependent host defence proteins such as myeloperoxidase.^125^ However, enterobactin can be sequestered by human LCN2, limiting its efficacy. LCN2 restricts the growth of bacteria that rely solely on enterobactin in a number of *in vivo* and *in vitro* models.^126^, ^127^, ^128^, ^129^

Demonstrating the strong selective pressure that LCN2 has placed on pathogenic bacteria, *Salmonella* also produces salmochelin, a glucosylated form of enterobactin that avoids capture by LCN2. Salmochelin is synthesized and mobilized by the *Fur*‐regulated tandem *iroN iroABCDE* gene cluster.^130^, ^131^ *Salmonella *Typhimurium and *S. *Typhi defective in enterobactin or salmochelin production or handling exhibit attenuated virulence, demonstrating the importance of both siderophore systems.^118^, ^119^, ^132^ In particular, salmochelin facilitates pathogen iron acquisition and proliferation in infectious situations where inflammation induces LCN2 expression.^133^ In addition to producing enterobactin and salmochelin, *Salmonella* also has the capacity to pirate exogenous siderophores produced by other bacteria and fungi, such as the fungal siderophore ferrichrome – a hydroxamate siderophore.^124^ However, the role of this mechanism during infection is still unclear.^134^

The genome of *C. difficile* encodes several ferritin‐, ferric‐, and siderophore‐bound iron uptake systems, which are almost exclusively Fur‐regulated and highly responsive to iron fluctuations.^91^, ^135^ Previously, it was shown that the catecholate’s precursor spermidine biosynthetic‐ and transport‐coding genes (*speAHEB* and *potABCD*, respectively), as well as the catecholate siderophore import system YclNOPQ, are Fur‐regulated and highly induced in *C. difficile* grown under iron‐limiting conditions.^91^, ^136^ Moreover, iron deprivation also induced expression of putative ferric hydroxamate uptake and sulphonate ABC transporter systems, which might facilitate iron uptake during iron‐poor conditions.^91^, ^92^

*Clostridium difficile* has been hypothesized to import siderophores produced by other microorganisms, but not to produce them. However, a recent study conducted by Shaw *et al*.^137^ identified a large genetic island from the genome of clade 3‐associated strains of *C. difficile*, which, among other peptides, is predicted to code for the biosynthesis of a Yersiniabactin‐like siderophore.

### Extraction and capture of haem

Due to the relative abundance of haem *in vivo*, and the insolubility of ferric iron, invading bacteria have evolved several mechanisms to access it from dietary sources, intracellular haemoproteins, or circulating haemoglobin. Haemoglobin – or spontaneously dissociated haem/haemin – may be captured directly by bacterial cell surface‐associated haemoglobin/haem‐binding transporters, or acquired indirectly via small soluble high‐affinity haem‐binding proteins called haemophores.^138^ While pathogenic strains of *E. coli*, *Yersinia* and *Pseudomonas* all express haem acquisition systems that seem to play a role in virulence, the importance of haem as a nutrient source for *Salmonella* remains unclear.^139^ Nevertheless, both salmolysin and the Typhi‐specific haemolysin E are proposed as being required for systemic infection.^140^, ^141^ In other bacterial infections, haemolysin‐mediated lysis of red blood cells may facilitate iron acquisition and infection. In response, host scavenging of free haem and haemoglobin by the acute‐phase proteins haemopexin and haptoglobin, respectively, is another aspect of reactive nutritional immunity.^142^

*Clostridium difficile* infection is characterized by high levels of luminal haemoglobin from lysed erythrocytes. Until now, haem uptake mechanisms remained elusive in *C. difficile*. Bioinformatic analyses of *C. difficile*'s genome unravelled an incomplete haem biosynthetic pathway, despite the presence of both sirohaem and cobalamine biosynthetic genes.^143^, ^144^ Recently, Knippel *et al*.^145^ identified a haem‐sensing membrane protein system (HsmRA), able to hijack and internalize haem from the host, using it as a defence mechanism against ROS generated by extracellular haemoglobin and innate immune cells. By sensing low haem concentrations, HsmR activates expression of the operon *hsmRA*; this prompts the binding of haem to the membrane‐bound HsmA, which, due to haem’s reactivity, is able to shield the bacterium from redox‐active species. Simultaneously, the haem activated transporter system complex patrols the intracellular environment for toxic levels of haem, by allowing its export when deemed necessary.^146^ It remains unclear to what extent *C. difficile*’s toxin‐driven tissue destruction and haemolysis may facilitate iron uptake; however, cholera toxin was recently shown to produce a growth‐enhancing effect on *Vibrio cholerae* growth in the gut lumen by facilitating iron acquisition.^147^

### Gut microbiota

The human gastrointestinal tract harbours a complex and dynamic collection of microorganisms, collectively entitled the gut microbiota. Microbial composition undergoes temporal and spatial variations as a result of nutrient availability caused by environmental factors such as age, health status, dietary changes, inflammation, pathogen and/or antibiotic exposure.^148^, ^149^ The mucosal intestinal immune system is generally tolerogenic, allowing the survival and proliferation of commensal microbes in exchange for immune and metabolic homeostasis and protection against pathogens.^148^, ^149^, ^150^ Therefore, an unfavourable compositional or functional change within the microbiome (dysbiosis) can incite infection, acute inflammatory responses and even chronic diseases such as inflammatory bowel disease and colorectal cancer.^151^

### Iron availability modulates the microbiota

The specific hallmarks of iron homeostasis in the 2000–4000 bacterial strains that constitute the gut microbiota remain to be described. Iron supplementation trials in infants in low‐ to middle‐income countries showed increased incidence of diarrhoeal disease.^152^, ^153^, ^154^, ^155^ This has been linked to higher levels of luminal iron influencing the composition of the gut microbiota.^156^ Althuogh complex, one consistent result from *in vivo* studies is an iron‐induced skew away from commensals such as lactobacilli or *Bifidobacterium* species,^154^, ^157^, ^158^ which have low iron requirements,^53^ towards *Enterobacteriaceae* (the bacterial family which includes *Salmonella* sp.). This skew and associated gut inflammation were particularly strong in low‐ to middle‐income country cohorts, probably because relatively poor sanitary conditions increase the risk of underlying colonization by pathogenic microorganisms, the proliferation of which may be facilitated by extra iron availability.^156^ Indeed, iron availability in the colon lumen influences the expression of pathogen virulent genes. FPN‐mediated effluxes of iron were shown to favour the growth of *S*. Typhimurium, conferring on them an advantage in the invasion of epithelial cells.^81^, ^159^ Also, a recent study demonstrated that gut inflammation – and associated stressors, like production of reactive oxygen intermediates and reactive nitrogen intermediates – stimulate *Salmonella*’s SOS response, which triggers bacteriophage lytic induction and transfer, thereby boosting horizontal gene transfer among bacteria and reassortment of their virulence factors.^160^ Whether iron supplementation alters the evolution of virulent bacterial strains remains to be investigated.

Experiments in animal models and *in vitro* fermentation systems broadly support the claims that iron supplementation remodels the gut microbiota,^161^, ^162^ potentially skewing towards a dysbiotic phenotype. However, some studies suggest that profound iron limitation may also disrupt the normal microbiota.^163^, ^164^ Although the picture is in no way clear‐cut, it seems that increasing iron availability in the gastrointestinal tract probably alters both the composition and metabolic activity of the gut microbiota, creating a niche through which pathogenic microorganisms can enter and cause disease.

### Gut microbiota modulates iron uptake and systemic iron homeostasis

Comparisons of germ‐free and microbiota‐colonized mice indicate that microbial colonization reduces luminal iron in the caecum.^165^ Conversely, when placed on a low‐iron diet, germ‐free mice exhibited milder iron‐deficiency‐induced anaemia, perhaps suggesting more efficient iron uptake in the absence of competing microbiota.^166^ The gut microbiota, specifically *Lactobacillus* sp., were found to reduce host iron uptake through the production of small molecule metabolites, including the 1,3‐diaminopropane, which inhibited HIF2*α* and expression of iron uptake proteins in the enterocyte. 1,3‐Diaminopropane production by lactobacilli was specifically enhanced during dietary iron deficiency, highlighting how the microbiota modulates local host iron homeostasis and luminal iron availability.^166^ Mouse models also suggest that, through its capacity to modulate systemic inflammatory responses, the gut microbiota can determine the extent of hepcidin induction and systemic hypoferraemia.^167^

### Commensal gut microbiota and iron acquisition by pathogenic microorganisms

The microbiota‐enforced status quo protects against gastrointestinal infection, as demonstrated by antibiotic therapy increasing the risk of *C. difficile* and *Salmonella* infections, whereas limitation of nutrient availability by the microbiota also plays a role.^149^ Specific strains of commensal bacteria are particularly adept at competing with pathogens for iron, even under inflammatory conditions when host‐imposed iron limitation ‐ through increased production of LCN2 and Lf – can disadvantage the microbiota as a whole. The non‐pathogenic *E. coli* strain Nissle has complex iron acquisition pathways and can secrete and utilize not only enterobactin, but also LCN2‐resistant salmochelin, yersiniabactin and aerobactin to effectively acquire iron at the expense of *Salmonella*.^17^, ^168^, ^169^ Furthermore, *E. coli* strain Nissle targets the uptake of siderophore‐bound iron by pathogenic *Enterobacteriaceae* – such as *Salmonella* – by secreting microcins, small anti‐bacterial molecules that bind bacteria via their catecholate siderophore receptors.^170^ *Bifidobacteria* strains with high iron sequestration properties have also been proposed to antagonize *S*. Typhi and pathogenic *E. coli in vitro*.^171^ Although bacterial iron homeostasis in single‐species situations is relatively well understood, our understanding of iron homeostasis in the multi‐species interactions within the gut remains in its infancy.^124^

## Future perspectives: therapeutic opportunities

### Targeting the pathogen

Iron uptake in the face of host and commensal defences is a major requirement for pathogen virulence, with iron acquisition systems being ubiquitous features of pathogenicity islands within genomes of virulent bacteria.^172^ However, although pathogens seem to suffer from iron deprivation, the dependence of innate and adaptive cellular immunity on iron makes therapeutically targeting iron availability problematic.^173^

An alternative approach to targeting iron directly may be to exploit our growing knowledge of pathogen iron uptake mechanisms. Improving host nutritional immunity through immunization against enterobactins reduced the severity of experimental gastrointestinal *Salmonella* infection, systemic dissemination and infection‐associated dysbiosis. Importantly, this approach supported, rather than undermined, the non‐pathogenic role of commensal microbiota.^174^ Alternatively, some investigators have proposed mirroring the mechanism of action of bacterially produced microcins and sideromycins, and exploiting siderophores as vehicles to facilitate the delivery of conjugated antibiotics to pathogens, overcoming resistance mechanisms such as enhanced drug efflux activity.^170^, ^175^ Finally, siderophore‐based therapies extend to exploiting probiotic bacteria that can more effectively compete with *Salmonella* for iron.^169^ These concepts of manipulating commensal–pathogen competition for nutrients, such as iron, may also prove relevant to *C. difficile* infection, where faecal microbial transfer has had therapeutic success through normalization of the commensal microbiota.^149^, ^176^

### Targeting the host

Probiotics and prebiotics may be useful in the treatment of iron deficiency and iron overload, by altering bioavailability of iron in the gastrointestinal tract and iron uptake by the enterocyte.^166^, ^177^, ^178^, ^179^ Furthermore, dysbiosis in gastrointestinal and systemic auto‐inflammatory disorders may in turn influence systemic iron metabolism, contributing to the anaemia of inflammation and functional iron deficiency commonly observed in these conditions.^151^, ^167^, ^180^

On the flip side, the potential for dysbiosis driven by dietary iron supplementation, thus exacerbating disease, complicates the treatment of anaemia in inflammatory bowel disease patients.^181^ A similar consideration must be made regarding the use of dietary iron supplementation to treat anaemia in low‐ to moderate‐income countries, where aggravating already high rates of infant diarrhoea is a clear risk; reducing inflammation and improving sanitary conditions will be instrumental if oral iron supplementation to treat anaemia is to prove fruitful.^156^, ^182^ Impaired gut health is a common reason for poor compliance with dietary iron supplementation regimens, even in high‐income countries.^183^ There is interest in developing dietary iron supplements that are bioavailable to the host but not the microbiota, hopefully avoiding dysbiosis.^184^ By changing iron dosing schedules to reflect the negative feedback mechanisms intrinsic to mammalian iron homeostasis, fractional iron uptake can be maximized.^185^, ^186^ This may also limit the adverse effects of iron supplementation on the gut.

Finally, chronically high dietary iron and haem, often associated with diets rich in red meat, have been proposed to predispose towards inflammatory bowel disease and colorectal cancer,^187^ both via direct effects on the epithelium and promoting dysbiosis.^188^, ^189^ Conversely, iron‐deficient diets in experimental systems seem to favour the growth of protective microorganisms.^107^, ^166^

## Conclusions

Contemplating the role of iron provides some new insights into host–pathogen relationships in enteric infections, such as those involving *Salmonella* and *C. difficile*. Mechanisms of mammalian iron homeostasis and bacterial iron acquisition are well studied, at least in defined experimental systems. However, the interaction of iron with complex and diverse commensal and pathogenic gut microbial communities in the context of human disease still remains relatively uncharacterized. Targeting bacteria‐specific iron acquisition apparatus and promoting commensal iron uptake at the expense of pathogens, while optimizing iron availability to the host, may together foster improved therapeutic approaches to gastrointestinal infectious and inflammatory disorders.

## Disclosures

None of the authors have any conflicts of interest.

## Data Availability

Data sharing is not applicable to this article as no new data were created or analysed in this study.

## References

[1] Drakesmith H , Prentice A . Viral infection and iron metabolism. Nat Rev Microbiol 2008; 6:541–52. 18552864 10.1038/nrmicro1930

[2] Poulos TL . Heme enzyme structure and function. Chem Rev 2014; 114:3919–62. 24400737 10.1021/cr400415k PMC3981943

[3] Sharp P , Srai SK . Molecular mechanisms involved in intestinal iron absorption. World J Gastroenterol 2007; 13:4716–24. 17729393 10.3748/wjg.v13.i35.4716 PMC4611193

[4] Muchowska KB , Varma SJ , Moran J . Synthesis and breakdown of universal metabolic precursors promoted by iron. Nature 2019; 569:104. 31043728 10.1038/s41586-019-1151-1 PMC6517266

[5] Rouault TA . Iron‐sulfur proteins hiding in plain sight. Nat Chem Biol 2015; 11:442–5. 26083061 10.1038/nchembio.1843

[6] Gari K , León Ortiz AM , Borel V , Flynn H , Skehel JM , Boulton SJ . MMS19 links cytoplasmic iron‐sulfur cluster assembly to DNA metabolism. Science 2012; 337:243–5. 22678361 10.1126/science.1219664

[7] Thelander L , Gräslund A , Thelander M . Continual presence of oxygen and iron required for mammalian ribonucleotide reduction: possible regulation mechanism. Biochem Biophys Res Commun 1983; 110:859–65. 6340669 10.1016/0006-291x(83)91040-9

[8] Loenarz C , Schofield CJ . Expanding chemical biology of 2‐oxoglutarate oxygenases. Nat Chem Biol 2008; 4:152–6. 18277970 10.1038/nchembio0308-152

[9] Drakesmith H , Nemeth E , Ganz T . Ironing out ferroportin. Cell Metab 2015; 22:777–87. 26437604 10.1016/j.cmet.2015.09.006 PMC4635047

[10] Takeuchi K , Bjarnason I , Laftah AH , Latunde‐Dada GO , Simpson RJ , McKie AT . Expression of iron absorption genes in mouse large intestine. Scand J Gastroenterol 2005; 40:169–77. 15764147 10.1080/00365520510011489

[11] Hurrell R , Egli I . Iron bioavailability and dietary reference values. Am J Clin Nutr 1461S; 91:1461S–7. 10.3945/ajcn.2010.28674F 20200263

[12] Fleming MD , Trenor CC , Su MA , Foernzler D , Beier DR , Dietrich WF *et al* Microcytic anaemia mice have a mutation in Nramp2, a candidate iron transporter gene. Nat Genet 1997; 16:383–6. 9241278 10.1038/ng0897-383

[13] Gunshin H , Fujiwara Y , Custodio AO , Direnzo C , Robine S , Andrews NC . Slc11a2 is required for intestinal iron absorption and erythropoiesis but dispensable in placenta and liver. J Clin Invest 2005; 115:1258–66. 15849611 10.1172/JCI24356 PMC1077176

[14] McKie AT , Barrow D , Latunde‐Dada GO , Rolfs A , Sager G , Mudaly E *et al* An iron‐regulated ferric reductase associated with the absorption of dietary iron. Science 2001; 291:1755–9. 11230685 10.1126/science.1057206

[15] Gunshin H , Starr CN , Direnzo C , Fleming MD , Jin J , Greer EL *et al* Cybrd1 (duodenal cytochrome *b*) is not necessary for dietary iron absorption in mice. Blood 2005; 106:2879–83. 15961514 10.1182/blood-2005-02-0716 PMC1895297

[16] Jacobs A , Miles PM . Intraluminal transport of iron from stomach to small‐intestinal mucosa. Br Med J 1969; 4:778–81. 4243302 10.1136/bmj.4.5686.778 PMC1630276

[17] Glover J , Jacobs A . Observations on iron in the jejunal lumen after a standard meal. Gut 1971; 12:369–71. 4255818 10.1136/gut.12.5.369 PMC1411612

[18] Shawki A , Engevik MA , Kim RS , Knight PB , Baik RA , Anthony SR *et al* Intestinal brush‐border Na^+^/H^+^ exchanger‐3 drives H^+^‐coupled iron absorption in the mouse. Am J Physiol Gastrointest Liver Physiol 2016; 311:G423–30. 27390324 10.1152/ajpgi.00167.2016 PMC5076011

[19] White C , Yuan X , Schmidt PJ , Bresciani E , Samuel TK , Campagna D *et al* HRG1 is essential for heme transport from the phagolysosome of macrophages during erythrophagocytosis. Cell Metab 2013; 17:261–70. 23395172 10.1016/j.cmet.2013.01.005 PMC3582031

[20] Weintraub LR , Weinstein MB , Huser HJ , Rafal S . Absorption of hemoglobin iron: the role of a heme‐splitting substance in the intestinal mucosa. J Clin Invest 1968; 47:531–9. 5637141 10.1172/JCI105749 PMC297199

[21] Abboud S , Haile DJ . A novel mammalian iron‐regulated protein involved in intracellular iron metabolism. J Biol Chem 2000; 275:19906–12. 10747949 10.1074/jbc.M000713200

[22] Donovan A , Brownlie A , Zhou Y , Shepard J , Pratt SJ , Moynihan J *et al* Positional cloning of zebrafish ferroportin1 identifies a conserved vertebrate iron exporter. Nature 2000; 403:776–81. 10693807 10.1038/35001596

[23] Donovan A , Lima CA , Pinkus JL , Pinkus GS , Zon LI , Robine S *et al* The iron exporter ferroportin/Slc40a1 is essential for iron homeostasis. Cell Metab 2005; 1:191–200. 16054062 10.1016/j.cmet.2005.01.003

[24] McKie AT , Marciani P , Rolfs A , Brennan K , Wehr K , Barrow D *et al* A novel duodenal iron‐regulated transporter, IREG1, implicated in the basolateral transfer of iron to the circulation. Mol Cell 2000; 5:299–309. 10882071 10.1016/s1097-2765(00)80425-6

[25] Fuqua BK , Lu Y , Darshan D , Frazer DM , Wilkins SJ , Wolkow N *et al* The multicopper ferroxidase hephaestin enhances intestinal iron absorption in mice. PLoS One 2014; 9:e98792. 24896847 10.1371/journal.pone.0098792 PMC4045767

[26] Vulpe CD , Kuo YM , Murphy TL , Cowley L , Askwith C , Libina N *et al* Hephaestin, a ceruloplasmin homologue implicated in intestinal iron transport, is defective in the sla mouse. Nat Genet 1999; 21:195–9. 9988272 10.1038/5979

[27] Lim D , Kim KS , Jeong J‐H , Marques O , Kim H‐J , Song M *et al* The hepcidin‐ferroportin axis controls the iron content of *Salmonella*‐containing vacuoles in macrophages. Nat Commun 2018; 9:1–12. 29844422 10.1038/s41467-018-04446-8 PMC5974375

[28] Levy JE , Jin O , Fujiwara Y , Kuo F , Andrews NC . Transferrin receptor is necessary for development of erythrocytes and the nervous system. Nat Genet 1999; 21:396–9. 10192390 10.1038/7727

[29] Ohgami RS , Campagna DR , McDonald A , Fleming MD . The Steap proteins are metalloreductases. Blood 2006; 108:1388–94. 16609065 10.1182/blood-2006-02-003681 PMC1785011

[30] Muckenthaler MU , Rivella S , Hentze MW , Galy B . A red carpet for iron metabolism. Cell 2017; 168:344–61. 28129536 10.1016/j.cell.2016.12.034 PMC5706455

[31] Patel SJ , Frey AG , Palenchar DJ , Achar S , Bullough KZ , Vashisht A *et al* A PCBP1‐BolA2 chaperone complex delivers iron for cytosolic [2Fe‐2S] cluster assembly. Nat Chem Biol 2019; 15:872–81. 31406370 10.1038/s41589-019-0330-6 PMC6702080

[32] Nandal A , Ruiz JC , Subramanian P , Ghimire‐Rijal S , Sinnamon RA , Stemmler TL *et al* Activation of the HIF prolyl hydroxylase by the iron chaperones PCBP1 and PCBP2. Cell Metab 2011; 14:647–57. 22055506 10.1016/j.cmet.2011.08.015 PMC3361910

[33] Brzóska K , Meczyńska S , Kruszewski M . Iron‐sulfur cluster proteins: electron transfer and beyond. Acta Biochim Pol 2006; 53:685–91. 17143336

[34] Arosio P , Carmona F , Gozzelino R , Maccarinelli F , Poli M . The importance of eukaryotic ferritins in iron handling and cytoprotection. Biochem J 2015; 472:1–15. 26518749 10.1042/BJ20150787

[35] Mancias JD , Pontano Vaites L , Nissim S , Biancur DE , Kim AJ , Wang X *et al* Ferritinophagy via NCOA4 is required for erythropoiesis and is regulated by iron dependent HERC2‐mediated proteolysis. eLife 2015; 4:e10308 10.7554/eLife.10308 PMC4592949 26436293

[36] Quiles del Rey M , Mancias JD . NCOA4‐mediated ferritinophagy: a potential link to neurodegeneration. Front Neurosci 2019; 13:238 10.3389/fnins.2019.00238 30930742 PMC6427834

[37] Nemeth E , Ganz T . Regulation of iron metabolism by Hepcidin. Annu Rev Nutr 2006; 26:323–42. 16848710 10.1146/annurev.nutr.26.061505.111303

[38] Pigeon C , Ilyin G , Courselaud B , Leroyer P , Turlin B , Brissot P *et al* A new mouse liver‐specific gene, encoding a protein homologous to human antimicrobial peptide hepcidin, is overexpressed during iron overload. J Biol Chem 2001; 276:7811–9. 11113132 10.1074/jbc.M008923200

[39] Nicolas G , Bennoun M , Devaux I , Beaumont C , Grandchamp B , Kahn A *et al* Lack of hepcidin gene expression and severe tissue iron overload in upstream stimulatory factor 2 (USF2) knockout mice. Proc Natl Acad Sci USA 2001; 98:8780–5. 11447267 10.1073/pnas.151179498 PMC37512

[40] Roetto A , Papanikolaou G , Politou M , Alberti F , Girelli D , Christakis J *et al* Mutant antimicrobial peptide hepcidin is associated with severe juvenile hemochromatosis. Nat Genet 2003; 33:21–2. 12469120 10.1038/ng1053

[41] Rivera S , Nemeth E , Gabayan V , Lopez MA , Farshidi D , Ganz T . Synthetic hepcidin causes rapid dose‐dependent hypoferremia and is concentrated in ferroportin‐containing organs. Blood 2005; 106:2196–9. 15933050 10.1182/blood-2005-04-1766 PMC1895137

[42] Ganz T . Hepcidin and iron regulation, 10 years later. Blood 2011; 117:4425–33. 21346250 10.1182/blood-2011-01-258467 PMC3099567

[43] Ganz T , Nemeth E . Iron homeostasis in host defence and inflammation. Nat Rev Immunol 2015; 15:500–10. 26160612 10.1038/nri3863 PMC4801113

[44] Aschemeyer S , Qiao B , Stefanova D , Valore EV , Sek AC , Ruwe TA *et al* Structure‐function analysis of ferroportin defines the binding site and an alternative mechanism of action of hepcidin. Blood 2018; 131:899–910. 29237594 10.1182/blood-2017-05-786590 PMC5824336

[45] Qiao B , Sugianto P , Fung E , Del‐Castillo‐Rueda A , Moran‐Jimenez M‐J , Ganz T *et al* Hepcidin‐induced endocytosis of ferroportin is dependent on ferroportin ubiquitination. Cell Metab 2012; 15:918–24. 22682227 10.1016/j.cmet.2012.03.018 PMC3372862

[46] Mastrogiannaki M , Matak P , Delga S , Deschemin J‐C , Vaulont S , Peyssonnaux C . Deletion of HIF‐2*α* in the enterocytes decreases the severity of tissue iron loading in hepcidin knockout mice. Blood 2012; 119:587–90. 22128145 10.1182/blood-2011-09-380337

[47] Shah YM , Matsubara T , Ito S , Yim S‐H , Gonzalez FJ . Intestinal hypoxia‐inducible transcription factors are essential for iron absorption following iron deficiency. Cell Metab 2009; 9:152–64. 19147412 10.1016/j.cmet.2008.12.012 PMC2659630

[48] Taylor M , Qu A , Anderson ER , Matsubara T , Martin A , Gonzalez FJ *et al* Hypoxia‐inducible factor‐2*α* mediates the adaptive increase of intestinal ferroportin during iron deficiency in mice. Gastroenterology 2011; 140:2044–55. 21419768 10.1053/j.gastro.2011.03.007 PMC3109109

[49] Schwartz AJ , Das NK , Ramakrishnan SK , Jain C , Jurkovic MT , Wu J *et al* Hepatic hepcidin/intestinal HIF‐2*α* axis maintains iron absorption during iron deficiency and overload. J Clin Invest 2019; 129:336–48. 30352047 10.1172/JCI122359 PMC6307944

[50] Wilkinson N , Pantopoulos K . The IRP/IRE system *in vivo*: insights from mouse models. Front Pharmacol 2014; 5:176. 25120486 10.3389/fphar.2014.00176 PMC4112806

[51] Galy B , Ferring‐Appel D , Becker C , Gretz N , Gröne H‐J , Schümann K *et al* Iron regulatory proteins control a mucosal block to intestinal iron absorption. Cell Rep 2013; 3:844–57. 23523353 10.1016/j.celrep.2013.02.026

[52] Vanoaica L , Darshan D , Richman L , Schümann K , Kühn LC . Intestinal ferritin H is required for an accurate control of iron absorption. Cell Metab 2010; 12:273–82. 20816093 10.1016/j.cmet.2010.08.003

[53] Weinberg ED . The *Lactobacillus* anomaly: total iron abstinence. Perspect Biol Med 1997; 40:578–83. 9269745 10.1353/pbm.1997.0072

[54] Posey JE , Gherardini FC . Lack of a role for iron in the Lyme disease pathogen. Science 2000; 288:1651–3. 10834845 10.1126/science.288.5471.1651

[55] Escolar L , Pérez‐Martín J , de Lorenzo V . Opening the iron box: transcriptional metalloregulation by the Fur protein. J Bacteriol 1999; 181:6223–9. 10515908 10.1128/jb.181.20.6223-6229.1999 PMC103753

[56] Hantke K . Iron and metal regulation in bacteria. Curr Opin Microbiol 2001; 4:172–7. 11282473 10.1016/s1369-5274(00)00184-3

[57] Carpenter BM , Whitmire JM , Merrell DS . This is not your mother’s repressor: the complex role of fur in pathogenesis. Infect Immun 2009; 77:2590–601. 19364842 10.1128/IAI.00116-09 PMC2708581

[58] Fillat MF . The FUR (ferric uptake regulator) superfamily: diversity and versatility of key transcriptional regulators. Arch Biochem Biophys 2014; 546:41–52. 24513162 10.1016/j.abb.2014.01.029

[59] White A , Ding X , vanderSpek JC , Murphy JR , Ringe D . Structure of the metal‐ion‐activated diphtheria toxin repressor/ tox operator complex. Nature 1998; 394:502–6. 9697776 10.1038/28893

[60] Baichoo N , Helmann JD . Recognition of DNA by fur: a reinterpretation of the Fur box consensus sequence. J Bacteriol 2002; 184:5826–32. 12374814 10.1128/JB.184.21.5826-5832.2002 PMC135393

[61] Andrews SC , Robinson AK , Rodríguez‐Quiñones F . Bacterial iron homeostasis. FEMS Microbiol Rev 2003; 27:215–37. 12829269 10.1016/S0168-6445(03)00055-X

[62] Troxell B , Hassan HM . Transcriptional regulation by Ferric Uptake Regulator (Fur) in pathogenic bacteria. Front Cell Infect Microbiol 2013; 3:59. 24106689 10.3389/fcimb.2013.00059 PMC3788343

[63] Pham OH , McSorley SJ . Protective host immune responses to *Salmonella* infection. Future Microbiol 2015; 10:101–10. 25598340 10.2217/fmb.14.98 PMC4323267

[64] Jajere SM . A review of *Salmonella enterica* with particular focus on the pathogenicity and virulence factors, host specificity and antimicrobial resistance including multidrug resistance. Vet World 2019; 12:504–21. 31190705 10.14202/vetworld.2019.504-521 PMC6515828

[65] Dougan G , Baker S . *Salmonella enterica* serovar Typhi and the pathogenesis of typhoid fever. Annu Rev Microbiol 2014; 68:317–36. 25208300 10.1146/annurev-micro-091313-103739

[66] Ilyas B , Tsai CN , Coombes BK . Evolution of *Salmonella*–Host cell interactions through a dynamic bacterial genome. Front Cell Infect Microbiol 2017; 7:428 10.3389/fcimb.2017.00428 29034217 PMC5626846

[67] Karen CC , John GB . Biology of *Clostridium difficile*: implications for epidemiology and diagnosis. Annu Rev Microbiol 2011; 65:501–21. 21682645 10.1146/annurev-micro-090110-102824

[68] McDonald LC , Gerding DN , Johnson S , Bakken JS , Carroll KC , Coffin SE *et al* Clinical Practice Guidelines for *Clostridium difficile* Infection in Adults and Children: 2017 Update by the Infectious Diseases Society of America (IDSA) and Society for Healthcare Epidemiology of America (SHEA). Clin Infect Dis 2018; 66:e1–48. 29462280 10.1093/cid/cix1085 PMC6018983

[69] Bjarnason J , Southward CM , Surette MG . Genomic profiling of iron‐responsive genes in *Salmonella enterica* Serovar Typhimurium by high‐throughput screening of a random promoter library. J Bacteriol 2003; 185:4973–82. 12897017 10.1128/JB.185.16.4973-4982.2003 PMC166456

[70] Oglesby‐Sherrouse AG , Murphy ER . Iron‐responsive bacterial small RNAs: variations on a theme. Metallomics 2013; 5:276–86. 23340911 10.1039/c3mt20224k PMC3612141

[71] Kim JN . Roles of two RyhB paralogs in the physiology of *Salmonella enterica* . Microbiol Res 2016; 186–187:146–52. 10.1016/j.micres.2016.04.004 27242152

[72] Troxell B , Fink RC , Porwollik S , McClelland M , Hassan HM . The Fur regulon in anaerobically grown *Salmonella enterica* sv. Typhimurium: identification of new Fur targets. BMC Microbiol 2011; 11:236. 22017966 10.1186/1471-2180-11-236 PMC3212961

[73] Halsey TA , Vazquez‐Torres A , Gravdahl DJ , Fang FC , Libby SJ . The Ferritin‐Like Dps protein is required for *Salmonella enterica* Serovar Typhimurium oxidative stress resistance and virulence. Infect Immun 2004; 72:1155–8. 14742565 10.1128/IAI.72.2.1155-1158.2004 PMC321587

[74] Velayudhan J , Castor M , Richardson A , Main‐Hester KL , Fang FC . The role of ferritins in the physiology of *Salmonella enterica* sv. Typhimurium: a unique role for ferritin B in iron‐sulphur cluster repair and virulence. Mol Microbiol 2007; 63:1495–507. 17302823 10.1111/j.1365-2958.2007.05600.x

[75] Groisman EA . The Pleiotropic two‐component regulatory system PhoP‐PhoQ. J Bacteriol 2001; 183:1835–42. 11222580 10.1128/JB.183.6.1835-1842.2001 PMC95077

[76] Teixidó L , Cortés P , Bigas A , Alvarez G , Barbé J , Campoy S . Control by Fur of the nitrate respiration regulators NarP and NarL in *Salmonella enterica* . Int Microbiol 2010; 13:33–9. 20890837 10.2436/20.1501.01.108

[77] Hall HK , Foster JW . The role of fur in the acid tolerance response of *Salmonella typhimurium* is physiologically and genetically separable from its role in iron acquisition. J Bacteriol 1996; 178:5683–91. 8824613 10.1128/jb.178.19.5683-5691.1996 PMC178407

[78] Teixidó L , Carrasco B , Alonso JC , Barbé J , Campoy S . Fur activates the expression of *Salmonella enterica* pathogenicity island 1 by directly interacting with the hilD operator in vivo and in vitro. PLoS One 2011; 6:e19711. 21573071 10.1371/journal.pone.0019711 PMC3089636

[79] Ellermeier JR , Slauch JM . Fur regulates expression of the *Salmonella* pathogenicity Island 1 Type III secretion system through HilD. J Bacteriol 2008; 190:476–86. 17993530 10.1128/JB.00926-07 PMC2223717

[80] Troxell B , Sikes ML , Fink RC , Vazquez‐Torres A , Jones‐Carson J , Hassan HM . Fur negatively regulates hns and is required for the expression of HilA and virulence in *Salmonella enterica* Serovar Typhimurium. J Bacteriol 2011; 193:497–505. 21075923 10.1128/JB.00942-10 PMC3019815

[81] Kortman GAM , Boleij A , Swinkels DW , Tjalsma H . Iron availability increases the pathogenic potential of *Salmonella typhimurium* and other enteric pathogens at the intestinal epithelial interface. PLoS One 2012; 7:e29968. 22272265 10.1371/journal.pone.0029968 PMC3260200

[82] Varghese S , Wu A , Park S , Imlay KRC , Imlay JA . Submicromolar hydrogen peroxide disrupts the ability of Fur protein to control free‐iron levels in *Escherichia coli* . Mol Microbiol 2007; 64:822–830. 17462026 10.1111/j.1365-2958.2007.05701.x PMC3048849

[83] Leclerc J‐M , Dozois CM , Daigle F . Role of the *Salmonella enterica* serovar Typhi Fur regulator and small RNAs RfrA and RfrB in iron homeostasis and interaction with host cells. Microbiology 2013; 159:591–602. 23306672 10.1099/mic.0.064329-0

[84] Curtiss R , Wanda S‐Y , Gunn BM , Zhang X , Tinge SA , Ananthnarayan V *et al* *Salmonella enterica* Serovar Typhimurium strains with regulated delayed attenuation *in vivo* . Infect Immun 2009; 77:1071–82. 19103774 10.1128/IAI.00693-08 PMC2643627

[85] Jones AM , Elliott T . A purified mutant HemA protein from *Salmonella enterica* serovar Typhimurium lacks bound heme and is defective for heme‐mediated regulation *in vivo* . FEMS Microbiol Lett 2010; 307:41–7. 20412302 10.1111/j.1574-6968.2010.01967.x

[86] Choby JE , Skaar EP . Heme synthesis and acquisition in bacterial pathogens. J Mol Biol 2016; 428:3408–28. 27019298 10.1016/j.jmb.2016.03.018 PMC5125930

[87] Elgrably‐Weiss M , Park S , Schlosser‐Silverman E , Rosenshine I , Imlay J , Altuvia S . A *Salmonella enterica* Serovar Typhimurium hemA mutant is highly susceptible to oxidative DNA damage. J Bacteriol 2002; 184:3774–84. 12081946 10.1128/JB.184.14.3774-3784.2002 PMC135181

[88] Wang LY , Brown L , Elliott M , Elliott T . Regulation of heme biosynthesis in *Salmonella typhimurium*: activity of glutamyl‐tRNA reductase (HemA) is greatly elevated during heme limitation by a mechanism which increases abundance of the protein. J Bacteriol 1997; 179:2907–14. 9139907 10.1128/jb.179.9.2907-2914.1997 PMC179053

[89] Benjamin WH , Hall P , Briles DE . A hemA mutation renders *Salmonella typhimurium* avirulent in mice, yet capable of eliciting protection against intravenous infection with *S. typhimurium* . Microb Pathog 1991; 11:289–95. 1813780 10.1016/0882-4010(91)90033-7

[90] Ho TD , Ellermeier CD . Ferric uptake regulator fur control of putative iron acquisition systems in *Clostridium difficile* . J Bacteriol 2015; 197:2930–40. 26148711 10.1128/JB.00098-15 PMC4542176

[91] Berges M , Michel A‐M , Lassek C , Nuss AM , Beckstette M , Dersch P *et al* Iron regulation in Clostridioides difficile. Front Microbiol 2018; 9:3183 10.3389/fmicb.2018.03183 30619231 PMC6311696

[92] Hastie JL , Hanna PC , Carlson PE . Transcriptional response of *Clostridium difficile* to low iron conditions. Pathog Dis 2018; 76:fty009 10.1093/femspd/fty009 PMC6251574 29390127

[93] Weinberg ED . Iron and susceptibility to infectious disease. Science 1974; 184:952–6. 4596821 10.1126/science.184.4140.952

[94] Nairz M , Schroll A , Haschka D , Dichtl S , Sonnweber T , Theurl I *et al* Lipocalin‐2 ensures host defense against *Salmonella* Typhimurium by controlling macrophage iron homeostasis and immune response. Eur J Immunol 2015; 45:3073–86. 10.1002/eji.201545569 26332507 PMC4688458

[95] Nairz M , Ferring‐Appel D , Casarrubea D , Sonnweber T , Viatte L , Schroll A *et al* Iron regulatory proteins mediate host resistance to Salmonella infection. Cell Host Microbe 2015; 18:254–61. 26190773 10.1016/j.chom.2015.06.017 PMC4666941

[96] Drakesmith H , Prentice AM . Hepcidin and the iron–infection axis. Science 2012; 338:768–72. 23139325 10.1126/science.1224577

[97] Park CH , Valore EV , Waring AJ , Ganz T . Hepcidin, a urinary antimicrobial peptide synthesized in the liver. J Biol Chem 2001; 276:7806–10. 11113131 10.1074/jbc.M008922200

[98] Nemeth E , Rivera S , Gabayan V , Keller C , Taudorf S , Pedersen BK *et al* IL‐6 mediates hypoferremia of inflammation by inducing the synthesis of the iron regulatory hormone hepcidin. J Clin Invest 2004; 113:1271–6. 15124018 10.1172/JCI20945 PMC398432

[99] Cross JH , Bradbury RS , Fulford AJ , Jallow AT , Wegmüller R , Prentice AM *et al* Oral iron acutely elevates bacterial growth in human serum. Sci Rep 2015; 5:16670. 26593732 10.1038/srep16670 PMC4655407

[100] Stefanova D , Raychev A , Arezes J , Ruchala P , Gabayan V , Skurnik M *et al* Endogenous hepcidin and its agonist mediate resistance to selected infections by clearing non‐transferrin‐bound iron. Blood 2017; 130:245–57. 28465342 10.1182/blood-2017-03-772715 PMC5520472

[101] Stefanova D , Raychev A , Deville J , Humphries R , Campeau S , Ruchala P *et al* Hepcidin protects against lethal *Escherichia coli* sepsis in mice inoculated with isolates from septic patients. Infect Immun 2018; 86:e00253‐18. 29735522 10.1128/IAI.00253-18 PMC6013672

[102] Arezes J , Jung G , Gabayan V , Valore E , Ruchala P , Gulig PA *et al* Hepcidin‐induced hypoferremia is a critical host defense mechanism against the siderophilic bacterium *Vibrio vulnificus* . Cell Host Microbe 2015; 17:47–57. 25590758 10.1016/j.chom.2014.12.001 PMC4296238

[103] Thwaites PA , Woods ML . Sepsis and siderosis, *Yersinia enterocolitica* and hereditary haemochromatosis. BMJ Case Rep 2017; 2017:bcr2016218185 10.1136/bcr-2016-218185 PMC5256388 28052950

[104] Nairz M , Haschka D , Demetz E , Weiss G . Iron at the interface of immunity and infection. Front Pharmacol 2014; 5:152 10.3389/fphar.2014.00152 25076907 PMC4100575

[105] Kim D‐K , Jeong J‐H , Lee J‐M , Kim KS , Park S‐H , Kim YD *et al* Inverse agonist of estrogen‐related receptor γ controls *Salmonella typhimurium* infection by modulating host iron homeostasis. Nat Med 2014; 20:419–24. 24658075 10.1038/nm.3483

[106] Zumerle S , Mathieu JRR , Delga S , Heinis M , Viatte L , Vaulont S *et al* Targeted disruption of hepcidin in the liver recapitulates the hemochromatotic phenotype. Blood 2014; 123:3646–50. 24646470 10.1182/blood-2014-01-550467

[107] Bessman NJ , Mathieu JRR , Renassia C , Zhou L , Fung TC , Fernandez KC *et al* Dendritic cell–derived hepcidin sequesters iron from the microbiota to promote mucosal healing. Science 2020; 368:186–9. 32273468 10.1126/science.aau6481 PMC7724573

[108] Kayama H , Kohyama M , Okuzaki D , Motooka D , Barman S , Okumura R *et al* Heme ameliorates dextran sodium sulfate‐induced colitis through providing intestinal macrophages with noninflammatory profiles. Proc Natl Acad Sci USA 2018; 115:8418–23. 30061415 10.1073/pnas.1808426115 PMC6099887

[109] Malerba M , Louis S , Cuvellier S , Shambat SM , Hua C , Gomart C *et al* Epidermal hepcidin is required for neutrophil response to bacterial infection. J Clin Invest 2020; 130:329–34. 31600168 10.1172/JCI126645 PMC6934188

[110] Wessling‐Resnick M . Nramp1 and other transporters involved in metal withholding during infection. J Biol Chem 2015; 290:18984–90. 26055722 10.1074/jbc.R115.643973 PMC4521020

[111] Li X , Yang Y , Zhou F , Zhang Y , Lu H , Jin Q *et al* SLC11A1 (NRAMP1) polymorphisms and tuberculosis susceptibility: updated systematic review and meta‐analysis. PLoS One 2011; 6:e15831. 21283567 10.1371/journal.pone.0015831 PMC3026788

[112] Forbes JR , Gros P . Iron, manganese, and cobalt transport by Nramp1 (Slc11a1) and Nramp2 (Slc11a2) expressed at the plasma membrane. Blood 2003; 102:1884–92. 12750164 10.1182/blood-2003-02-0425

[113] Jabado N , Jankowski A , Dougaparsad S , Picard V , Grinstein S , Gros P . Natural resistance to intracellular infections: natural resistance‐associated macrophage protein 1 (Nramp1) functions as a pH‐dependent manganese transporter at the phagosomal membrane. J Exp Med 2000; 192:1237–48. 11067873 10.1084/jem.192.9.1237 PMC2193348

[114] Cunrath O , Bumann D . Host resistance factor SLC11A1 restricts *Salmonella* growth through magnesium deprivation. Science 2019; 366:995–9. 31753999 10.1126/science.aax7898

[115] Barton CH , Whitehead SH , Blackwell JM . Nramp transfection transfers Ity/Lsh/Bcg‐related pleiotropic effects on macrophage activation: influence on oxidative burst and nitric oxide pathways. Mol Med 1995; 1:267–79. 8529105 PMC2229912

[116] Hood MI , Skaar EP . Nutritional immunity: transition metals at the pathogen–host interface. Nat Rev Microbiol 2012; 10:525–37. 22796883 10.1038/nrmicro2836 PMC3875331

[117] Lau CKY , Krewulak KD , Vogel HJ . Bacterial ferrous iron transport: the Feo system. FEMS Microbiol Rev 2016; 40:273–98. 26684538 10.1093/femsre/fuv049

[118] Nagy TA , Moreland SM , Detweiler CS . *Salmonella* acquires ferrous iron from hemophagocytic macrophages. Mol Microbiol 2014; 93:1314–26. 25081030 10.1111/mmi.12739 PMC4160465

[119] Tsolis RM , Bäumler AJ , Heffron F , Stojiljkovic I . Contribution of TonB‐ and Feo‐mediated iron uptake to growth of *Salmonella typhimurium* in the mouse. Infect Immun 1996; 64:4549–56. 8890205 10.1128/iai.64.11.4549-4556.1996 PMC174411

[120] Schröder I , Johnson E , de Vries S . Microbial ferric iron reductases. FEMS Microbiol Rev 2003; 27:427–47. 12829278 10.1016/S0168-6445(03)00043-3

[121] Nakashige TG , Zhang B , Krebs C , Nolan EM . Human calprotectin is an iron‐sequestering host‐defense protein. Nat Chem Biol 2015; 11:765–71. 26302479 10.1038/nchembio.1891 PMC4575267

[122] Legrand D , Elass E , Pierce A , Mazurier J . Lactoferrin and host defence: an overview of its immuno‐modulating and anti‐inflammatory properties. Biometals 2004; 17:225–9. 15222469 10.1023/b:biom.0000027696.48707.42

[123] Barber MF , Elde NC . Escape from bacterial iron piracy through rapid evolution of transferrin. Science 2014; 346:1362–6. 25504720 10.1126/science.1259329 PMC4455941

[124] Kramer J , Özkaya Ö , Kümmerli R . Bacterial siderophores in community and host interactions. Nat Rev Microbiol 2020; 18:152–63. 31748738 10.1038/s41579-019-0284-4 PMC7116523

[125] Singh V , Yeoh BS , Xiao X , Kumar M , Bachman M , Borregaard N *et al* Interplay between enterobactin, myeloperoxidase and lipocalin 2 regulates *E. coli* survival in the inflamed gut. Nat Commun 2015; 6:1–11. 10.1038/ncomms8113 PMC6336494 25964185

[126] Flo TH , Smith KD , Sato S , Rodriguez DJ , Holmes MA , Strong RK *et al* Lipocalin 2 mediates an innate immune response to bacterial infection by sequestrating iron. Nature 2004; 432:917–21. 15531878 10.1038/nature03104

[127] Yang J , Goetz D , Li J‐Y , Wang W , Mori K , Setlik D *et al* An iron delivery pathway mediated by a Lipocalin. Mol Cell 2002; 10:1045–56. 12453413 10.1016/s1097-2765(02)00710-4

[128] Moschen AR , Adolph TE , Gerner RR , Wieser V , Tilg H . Lipocalin‐2: a master mediator of intestinal and metabolic inflammation. Trends Endocrinol Metab 2017; 28:388–97. 28214071 10.1016/j.tem.2017.01.003

[129] Xiao X , Yeoh BS , Vijay‐Kumar M . Lipocalin 2: an emerging player in iron homeostasis and inflammation. Annu Rev Nutr 2017; 37:103–30. 28628361 10.1146/annurev-nutr-071816-064559

[130] Hantke K , Nicholson G , Rabsch W , Winkelmann G . Salmochelins, siderophores of *Salmonella enterica* and uropathogenic *Escherichia coli* strains, are recognized by the outer membrane receptor IroN. Proc Natl Acad Sci USA 2003; 100:3677–82. 12655053 10.1073/pnas.0737682100 PMC152981

[131] Fischbach MA , Lin H , Zhou L , Yu Y , Abergel RJ , Liu DR *et al* The pathogen‐associated iroA gene cluster mediates bacterial evasion of lipocalin 2. Proc Natl Acad Sci USA 2006; 103:16502–7. 17060628 10.1073/pnas.0604636103 PMC1637611

[132] Karlinsey JE , Stepien TA , Mayho M , Singletary LA , Bingham‐Ramos LK , Brehm MA *et al* Genome‐wide analysis of *Salmonella enterica* serovar Typhi in humanized mice reveals key virulence features. Cell Host Microbe 2019; 26: 426–34.e6. 31447308 10.1016/j.chom.2019.08.001 PMC6742556

[133] Raffatellu M , George MD , Akiyama Y , Hornsby MJ , Nuccio S‐P , Paixao TA *et al* Lipocalin‐2 resistance confers an advantage to *Salmonella enterica* serotype Typhimurium for growth and survival in the inflamed intestine. Cell Host Microbe 2009; 5:476–86. 19454351 10.1016/j.chom.2009.03.011 PMC2768556

[134] Kingsley RA , Reissbrodt R , Rabsch W , Ketley JM , Tsolis RM , Everest P *et al* Ferrioxamine‐mediated Iron(III) utilization by *Salmonella enterica* . Appl Environ Microbiol 1999; 65:1610–8. 10103258 10.1128/aem.65.4.1610-1618.1999 PMC91228

[135] Cernat RC , Scott KP . Evaluation of novel assays to assess the influence of different iron sources on the growth of *Clostridium difficile* . Anaerobe 2012; 18:298–304. 22554901 10.1016/j.anaerobe.2012.04.007

[136] Lopez CA , Beavers WN , Weiss A , Knippel RJ , Zackular JP , Chazin W *et al* The immune protein calprotectin impacts *Clostridioides difficile* metabolism through zinc limitation. mBio 2019; 10:e02289‐19 10.1128/mBio.02289-19 31744916 PMC6867894

[137] Shaw HA , Khodadoost L , Preston MD , Corver J , Mullany P , Wren BW . *Clostridium difficile* clade 3 (RT023) have a modified cell surface and contain a large transposable island with novel cargo. Sci Rep 2019; 9:15330. 31653906 10.1038/s41598-019-51628-5 PMC6814731

[138] Richard KL , Kelley BR , Johnson JG . Heme uptake and utilization by gram‐negative bacterial pathogens. Front Cell Infect Microbiol 2019; 9:81 10.3389/fcimb.2019.00081 30984629 PMC6449446

[139] Runyen‐Janecky LJ . Role and regulation of heme iron acquisition in gram‐negative pathogens. Front Cell Infect Microbiol 2013; 3:55 10.3389/fcimb.2013.00055 24116354 PMC3792355

[140] Libby SJ , Goebel W , Ludwig A , Buchmeier N , Bowe F , Fang FC *et al* A cytolysin encoded by *Salmonella* is required for survival within macrophages. Proc Natl Acad Sci 1994; 91:489–93. 8290552 10.1073/pnas.91.2.489 PMC42974

[141] Fuentes JA , Villagra N , Castillo‐Ruiz M , Mora GC . The *Salmonella* Typhi hlyE gene plays a role in invasion of cultured epithelial cells and its functional transfer to *S*. Typhimurium promotes deep organ infection in mice. Res Microbiol 2008; 159:279–87. 18434098 10.1016/j.resmic.2008.02.006

[142] Sakamoto K , Kim Y‐G , Hara H , Kamada N , Caballero‐Flores G , Tolosano E *et al* IL‐22 controls iron‐dependent nutritional immunity against systemic bacterial infections. Science immunology 2017; 2:eaai8371. 28286877 10.1126/sciimmunol.aai8371 PMC5345941

[143] Moore SJ , Warren MJ . The anaerobic biosynthesis of vitamin B12. Biochem Soc Trans 2012; 40:581–86. 22616870 10.1042/BST20120066

[144] Dailey HA , Dailey TA , Gerdes S , Jahn D , Jahn M , O'Brian MR *et al* Prokaryotic heme biosynthesis: multiple pathways to a common essential product. Microbiol Mol Biol Rev 2017; 81:e00048‐16 10.1128/MMBR.00048-16 28123057 PMC5312243

[145] Knippel RJ , Wexler AG , Miller JM , Beavers WN , Weiss A , de Crécy‐Lagard V *et al* *Clostridioides difficile* senses and Hijacks Host Heme for incorporation into an oxidative stress defense system. Cell Host Microbe 2020 10.1016/j.chom.2020.05.015 PMC7486240 32526159

[146] Knippel RJ , Zackular JP , Moore JL , Celis AI , Weiss A , Washington MK *et al* Heme sensing and detoxification by HatRT contributes to pathogenesis during *Clostridium difficile* infection. PLoS Pathog 2018; 14:e1007486. 30576368 10.1371/journal.ppat.1007486 PMC6303022

[147] Rivera‐Chávez F , Mekalanos JJ . Cholera toxin promotes pathogen acquisition of host‐derived nutrients. Nature 2019; 572:244–8. 31367037 10.1038/s41586-019-1453-3 PMC6727848

[148] Kolodziejczyk AA , Zheng D , Elinav E . Diet–microbiota interactions and personalized nutrition. Nat Rev Microbiol 2019; 17:742–53. 31541197 10.1038/s41579-019-0256-8

[149] Bäumler AJ , Sperandio V . Interactions between the microbiota and pathogenic bacteria in the gut. Nature 2016; 535:85–93. 27383983 10.1038/nature18849 PMC5114849

[150] Honda K , Littman DR . The microbiota in adaptive immune homeostasis and disease. Nature 2016; 535:75–84. 27383982 10.1038/nature18848

[151] Lynch SV , Pedersen O . The human intestinal microbiome in health and disease. N Engl J Med 2016; 375:2369–79. 27974040 10.1056/NEJMra1600266

[152] Soofi S , Cousens S , Iqbal SP , Akhund T , Khan J , Ahmed I *et al* Effect of provision of daily zinc and iron with several micronutrients on growth and morbidity among young children in Pakistan: a cluster‐randomised trial. Lancet 2013; 382:29–40. 23602230 10.1016/S0140-6736(13)60437-7

[153] Gera T , Sachdev HPS . Effect of iron supplementation on incidence of infectious illness in children: systematic review. BMJ 2002; 325:1142. 12433763 10.1136/bmj.325.7373.1142 PMC133452

[154] Jaeggi T , Kortman GAM , Moretti D , Chassard C , Holding P , Dostal A *et al* Iron fortification adversely affects the gut microbiome, increases pathogen abundance and induces intestinal inflammation in Kenyan infants. Gut 2015; 64:731–42. 25143342 10.1136/gutjnl-2014-307720

[155] Zimmermann MB , Chassard C , Rohner F , N’Goran EK , Nindjin C , Dostal A *et al* The effects of iron fortification on the gut microbiota in African children: a randomized controlled trial in Côte d’Ivoire. Am J Clin Nutr 2010; 92:1406–15. 20962160 10.3945/ajcn.110.004564

[156] Paganini D , Zimmermann MB . The effects of iron fortification and supplementation on the gut microbiome and diarrhea in infants and children: a review. Am J Clin Nutr 1688S; 106:1688S–93. 10.3945/ajcn.117.156067 PMC5701709 29070552

[157] Tang M , Frank DN , Hendricks AE , Ir D , Esamai F , Liechty E *et al* Iron in micronutrient powder promotes an unfavorable gut microbiota in Kenyan infants. Nutrients 2017; 9:776. 10.3390/nu9070776 PMC5537890 28753958

[158] Mevissen‐Verhage EA , Marcelis JH , Harmsen‐Van Amerongen WC , de Vos NM , Verhoef J . Effect of iron on neonatal gut flora during the first three months of life. Eur J Clin Microbiol 1985; 4:273–8. 3894015 10.1007/BF02013651

[159] Chlosta S , Fishman DS , Harrington L , Johnson EE , Knutson MD , Wessling‐Resnick M *et al* The iron efflux protein ferroportin regulates the intracellular growth of *Salmonella enterica* . Infect Immun 2006; 74:3065–7. 16622252 10.1128/IAI.74.5.3065-3067.2006 PMC1459754

[160] Diard M , Bakkeren E , Cornuault JK , Moor K , Hausmann A , Sellin ME *et al* Inflammation boosts bacteriophage transfer between *Salmonella* spp. Science 2017; 355:1211–5. 28302859 10.1126/science.aaf8451

[161] Kortman GAM , Mulder MLM , Richters TJW , Shanmugam NKN , Trebicka E , Boekhorst J *et al* Low dietary iron intake restrains the intestinal inflammatory response and pathology of enteric infection by food‐borne bacterial pathogens. Eur J Immunol 2015; 45:2553–67. 26046550 10.1002/eji.201545642 PMC4618841

[162] Kortman GAM , Dutilh BE , Maathuis AJH , Engelke UF , Boekhorst J , Keegan KP *et al* Microbial metabolism shifts towards an adverse profile with supplementary iron in the TIM‐2 *in vitro* model of the human colon. Front Microbiol 2016; 6:1481 10.3389/fmicb.2015.01481 26779139 PMC4701948

[163] Dostal A , Fehlbaum S , Chassard C , Zimmermann MB , Lacroix C . Low iron availability in continuous in vitro colonic fermentations induces strong dysbiosis of the child gut microbial consortium and a decrease of main metabolites. FEMS Microbiol Ecol 2013; 83:161–75. 22845175 10.1111/j.1574-6941.2012.01461.x PMC3511601

[164] Parmanand BA , Kellingray L , Le Gall G , Basit AW , Fairweather‐Tait S , Narbad A . A decrease in iron availability to human gut microbiome reduces the growth of potentially pathogenic gut bacteria; an *in vitro* colonic fermentation study. J Nutr Biochem 2019; 67:20–27. 30831460 10.1016/j.jnutbio.2019.01.010 PMC6546957

[165] Deschemin J‐C , Noordine M‐L , Remot A , Willemetz A , Afif C , Canonne‐Hergaux F *et al* The microbiota shifts the iron sensing of intestinal cells. FASEB J 2015; 30:252–61. 26370847 10.1096/fj.15-276840

[166] Das NK , Schwartz AJ , Barthel G , Inohara N , Liu Q , Sankar A *et al* Microbial metabolite signaling is required for systemic iron homeostasis. Cell Metab 2020; 31: 115–30.e6. 31708445 10.1016/j.cmet.2019.10.005 PMC6949377

[167] Shanmugam NKN , Trebicka E , Fu L , Shi HN , Cherayil BJ . Intestinal inflammation modulates expression of the iron‐regulating hormone hepcidin depending on erythropoietic activity and the commensal microbiota. J Immunol 2014; 193:1398–407. 24973448 10.4049/jimmunol.1400278 PMC4108560

[168] Valdebenito M , Crumbliss AL , Winkelmann G , Hantke K . Environmental factors influence the production of enterobactin, salmochelin, aerobactin, and yersiniabactin in *Escherichia coli* strain Nissle 1917. Int J Med Microbiol 2006; 296:513–20. 17008127 10.1016/j.ijmm.2006.06.003

[169] Deriu E , Liu JZ , Pezeshki M , Edwards RA , Ochoa RJ , Contreras H *et al* Probiotic bacteria reduce *Salmonella* Typhimurium intestinal colonization by competing for iron. Cell Host Microbe 2013; 14:26–37. 23870311 10.1016/j.chom.2013.06.007 PMC3752295

[170] Sassone‐Corsi M , Nuccio S‐P , Liu H , Hernandez D , Vu CT , Takahashi AA *et al* Microcins mediate competition among *Enterobacteriaceae* in the inflamed gut. Nature 2016; 540:280–3. 27798599 10.1038/nature20557 PMC5145735

[171] Vazquez‐Gutierrez P , de Wouters T , Werder J , Chassard C , Lacroix C . High iron‐sequestrating bifidobacteria inhibit enteropathogen growth and adhesion to intestinal epithelial cells *in vitro* . Front Microbiol 2016; 7:1480 10.3389/fmicb.2016.01480 27713730 PMC5031772

[172] Schmidt H , Hensel M . Pathogenicity islands in bacterial pathogenesis. Clin Microbiol Rev 2004; 17:14–56. 14726454 10.1128/CMR.17.1.14-56.2004 PMC321463

[173] Collins HL , Kaufmann SHE , Schaible UE . Iron chelation via deferoxamine exacerbates experimental salmonellosis via inhibition of the nicotinamide adenine dinucleotide phosphate oxidase‐dependent respiratory burst. J Immunol 2002; 168:3458–63. 11907105 10.4049/jimmunol.168.7.3458

[174] Sassone‐Corsi M , Chairatana P , Zheng T , Perez‐Lopez A , Edwards RA , George MD *et al* Siderophore‐based immunization strategy to inhibit growth of enteric pathogens. Proc Natl Acad Sci USA 2016; 113:13462–7. 27821741 10.1073/pnas.1606290113 PMC5127304

[175] Zheng T , Nolan EM . Enterobactin‐mediated delivery of *β*‐lactam antibiotics enhances antibacterial activity against pathogenic *Escherichia coli* . J Am Chem Soc 2014; 136:9677–91. 24927110 10.1021/ja503911p PMC4353011

[176] Seekatz AM , Young VB . *Clostridium difficile* and the microbiota. J Clin Invest 2014; 124:4182–9. 25036699 10.1172/JCI72336 PMC4191019

[177] Paganini D , Uyoga MA , Cercamondi CI , Moretti D , Mwasi E , Schwab C *et al* Consumption of galacto‐oligosaccharides increases iron absorption from a micronutrient powder containing ferrous fumarate and sodium iron EDTA: a stable‐isotope study in Kenyan infants. Am J Clin Nutr 2017; 106:1020–31. 28814396 10.3945/ajcn.116.145060

[178] Jeroense FMD , Michel L , Zeder C , Herter‐Aeberli I , Zimmermann MB . Consumption of galacto‐oligosaccharides increases iron absorption from ferrous fumarate: a stable iron isotope study in iron‐depleted young women. J Nutr 2019; 149:738–46. 31004135 10.1093/jn/nxy327

[179] Vonderheid SC , Tussing‐Humphreys L , Park C , Pauls H , OjiNjideka Hemphill N , LaBomascus B *et al* A systematic review and meta‐analysis on the effects of probiotic species on iron absorption and iron status. Nutrients 2019; 11:2938. 10.3390/nu11122938 PMC6949908 31816981

[180] Zhang X , Zhang D , Jia H , Feng Q , Wang D , Liang D *et al* The oral and gut microbiomes are perturbed in rheumatoid arthritis and partly normalized after treatment. Nat Med 2015; 21:895–905. 26214836 10.1038/nm.3914

[181] Stein J , Dignass AU . Management of iron deficiency anemia in inflammatory bowel disease – a practical approach. Ann Gastroenterol 2013; 26:104–13. 24714874 PMC3959949

[182] Pasricha S‐R , Armitage AE , Prentice AM , Drakesmith H . Reducing anaemia in low income countries: control of infection is essential. BMJ 2018; 362:k3165. 30068664 10.1136/bmj.k3165

[183] Tolkien Z , Stecher L , Mander AP , Pereira DIA , Powell JJ . Ferrous sulfate supplementation causes significant gastrointestinal side‐effects in adults: a systematic review and meta‐analysis. PLoS One 2015; 10:e0117383. 25700159 10.1371/journal.pone.0117383 PMC4336293

[184] Pereira DIA , Mohammed NI , Ofordile O , Camara F , Baldeh B , Mendy T *et al* A novel nano‐iron supplement to safely combat iron deficiency and anaemia in young children: the IHAT‐GUT double‐blind, randomised, placebo‐controlled trial protocol. Gates Open Res 2018; 2:48. 30569038 10.12688/gatesopenres.12866.2 PMC6266659

[185] Stoffel NU , Zeder C , Brittenham GM , Moretti D , Zimmermann MB . Iron absorption from supplements is greater with alternate day than with consecutive day dosing in iron‐deficient anemic women. Haematologica 2020; 105:1232–9. 31413088 10.3324/haematol.2019.220830 PMC7193469

[186] Moretti D , Goede JS , Zeder C , Jiskra M , Chatzinakou V , Tjalsma H *et al* Oral iron supplements increase hepcidin and decrease iron absorption from daily or twice‐daily doses in iron‐depleted young women. Blood 2015; 126:1981–9. 26289639 10.1182/blood-2015-05-642223

[187] Fiorito V , Chiabrando D , Petrillo S , Bertino F , Tolosano E . The multifaceted role of heme in cancer. Front Oncol 2020; 9:1540 10.3389/fonc.2019.01540 32010627 PMC6974621

[188] Ijssennagger N , Belzer C , Hooiveld GJ , Dekker J , van Mil SWC , Müller M *et al* Gut microbiota facilitates dietary heme‐induced epithelial hyperproliferation by opening the mucus barrier in colon. Proc Natl Acad Sci USA 2015; 112:10038–43. 26216954 10.1073/pnas.1507645112 PMC4538683

[189] Constante M , Fragoso G , Calvé A , Samba‐Mondonga M , Santos MM . Dietary heme induces gut dysbiosis, aggravates colitis, and potentiates the development of adenomas in mice. Front Microbiol 2017; 8:1809 10.3389/fmicb.2017.01809 28983289 PMC5613120

